# The Role of *Staphylococcus aureus* and Its Toxins in the Pathogenesis of Allergic Asthma

**DOI:** 10.3390/ijms24010654

**Published:** 2022-12-30

**Authors:** Ilka Jorde, Jens Schreiber, Sabine Stegemann-Koniszewski

**Affiliations:** Experimental Pneumology, Department of Pneumology, University Hospital Magdeburg/Medical Faculty, Health Campus Immunology, Infectiology and Inflammation (GC-I3), Otto-von-Guericke-University, 39120 Magdeburg, Germany

**Keywords:** allergic asthma, *Staphylococcus aureus*, *Staphylococcus aureus* enterotoxin B, serine protease-like protein, toxic-shock-syndrome toxin 1, staphylococcal protein A, allergen, microbial colonization, atopic diseases, toxin-specific IgE-sensitization

## Abstract

Bronchial asthma is one of the most common chronic diseases worldwide and affects more than 300 million patients. Allergic asthma affects the majority of asthmatic children as well as approximately 50% of adult asthmatics. It is characterized by a Th2-mediated immune response against aeroallergens. Many aspects of the overall pathophysiology are known, while the underlying mechanisms and predisposing factors remain largely elusive today. Over the last decade, respiratory colonization with *Staphylococcus aureus* (*S. aureus*), a Gram-positive facultative bacterial pathogen, came into focus as a risk factor for the development of atopic respiratory diseases. More than 30% of the world’s population is constantly colonized with *S. aureus* in their nasopharynx. This colonization is mostly asymptomatic, but in immunocompromised patients, it can lead to serious complications including pneumonia, sepsis, or even death. *S. aureus* is known for its ability to produce a wide range of proteins including toxins, serine-protease-like proteins, and protein A. In this review, we provide an overview of the current knowledge about the pathophysiology of allergic asthma and to what extent it can be affected by different toxins produced by *S. aureus.* Intensifying this knowledge might lead to new preventive strategies for atopic respiratory diseases.

## 1. Bronchial Asthma

Bronchial asthma is a chronic inflammatory condition of the airways that affects more than 300 million patients worldwide [1]. The main characteristics are airway hyperreactivity and increased mucus production, as well as structural changes in the airways [2]. Airway hyperreactivity displays a characteristic functional abnormality of asthma that is partly reversible with therapy. In patients with severe asthma, a progressive, persistent airflow limitation can develop [3,4]. Amplified mucus production occurs due to an increase in the number of goblet cells and the size of submucosal glands in the airway epithelium and can display another cause for airflow obstruction [5]. Structural changes include epithelial detachment, which is, among other findings, characterized by increased apoptosis in the bronchial epithelial cells of patients with asthma as compared to epithelial cells of normal controls [4,6]. Subepithelial fibrosis and an increase in airway smooth muscle mass, which is in part comprised of hyperplasia, are also common observations in asthmatic patients [4,7]. During an asthmatic attack or exacerbation, severe shortness of breath, chest tightness, coughing, and wheezing can occur due to airflow limitations [8].

Historically, bronchial asthma was discriminated into two subtypes: Non-allergic (intrinsic) and allergic (extrinsic). Approximately 10–33% of asthmatic patients suffer from non-allergic or intrinsic asthma. This form of asthma can be diagnosed by the presence of asthma symptoms despite a lack of specific IgE or a negative skin prick test for a variety of seasonal and perennial allergens. Intrinsic asthma is characterized by a female predominance, a more rapid and severe progression—in comparison to allergic asthma—and a later onset in life [9,10]. In some patients suffering from intrinsic asthma, a prevalence of neutrophils in the respiratory tract is observed [10,11,12]. Until today, there has been no directed therapy considered effective in this endotype [11,13].

The majority of children and approximately 50% of adult patients suffer from allergic asthma [14]. Allergic airway reactions are typically characterized by a T helper type 2 (Th2) cell-dominated immune response towards aeroallergens. This response includes the production of allergen-specific IgE antibodies, the release of Th2-inflammatory mediators such as interleukin-4 (IL-4), IL-5, and IL-13, and the recruitment and activation of mast cells, eosinophils, and basophils [15,16].

Nowadays, it is known that asthma must be divided into far more than two subtypes: There is, e.g., type 2-high or ultra-high and type 2-low asthma, which can, again, be subdivided into different phenotypes, depending on, e.g., inflammatory mediators or the age of onset. While type 2-high asthma is characterized by Th2-associated cytokines and type 2-ultra high asthma reflects a more severe form of the disease, the definition of type 2-low endotypes is more complex, since no respective biomarkers have been identified so far. Underlying pathophysiological mechanisms are used to define the different endotypes, leading to differences in responsiveness to common therapies. Consequently, all asthmatic patients with no type 2-high inflammation are included in this type-2 low endotype [17,18,19].

## 2. Risk Factors and Strategies of Prevention

As heterogeneous as bronchial asthma itself, the triggers and risk factors for developing this disease are just as variable. The most important aeroallergens that trigger IgE-mediated allergies include grass, tree, and herb pollen, as well as house dust mites, animal dander, and mold spores [20]. Nowadays, multiple environmental and genetic risk factors that further promote the development of asthma are known, and these are addressed in the following section.

Commonly known environmental risk factors for developing asthma are tobacco smoke and air pollution [21]. The association between active and passive smoking and a higher risk of suffering from asthma has been demonstrated in a number of studies [22,23,24,25,26]. Another important environmental risk factor for the development of asthma is microbes. Bacterial and viral infections can be important factors in asthma pathogenesis. Commonly identified viruses causing respiratory tract infections are, amongst others, human rhinoviruses, the respiratory syncytial virus (RSV), influenza and parainfluenza virus, and coronaviruses [27,28]. The underlying mechanisms remain elusive, but several studies have demonstrated a significant association between early-life viral lower respiratory tract infections and the development of childhood asthma [29,30]. Since fairly recently, it has been accepted that the airways are constantly colonized by microorganisms and that these interact with the immune system in multiple ways in health and disease [31,32]. In addition to viral infections, colonization with bacteria such as *Streptococcus pneumoniae, Haemophilus influenzae,* or *Moraxella catarrhalis* in early life is associated with a persistent wheezing phenotype and asthma diagnosis [33]. In recent decades, the colonization with *Staphylococcus aureus* (*S. aureus*) came into focus as a risk factor for the development of asthma, not only early in life but also in adulthood. Different studies show a significant association between *S. aureus* colonization and asthma prevalence [34,35]. A special role is ascribed to staphylococcal enterotoxins. Several studies showed enterotoxin-specific IgE-sensitization in asthmatic patients [36,37,38,39]. Even patients with severe asthma diagnosed as non-atopic frequently showed IgE-sensitization towards *S. aureus* enterotoxins [40]. In contrast to microbes considered risk factors for the development of asthma, there is the hygiene hypothesis stating that childhood infections and exposure to microbial antigens early in life present a strong negative correlation with allergies. Due to the increased use of antibiotics, improved hygiene, and urbanization, the prevalence of allergic diseases has been on the rise in western industrial countries [41,42].

Next to environmental factors, physical and psychological conditions such as obesity or stress can enhance the risk of developing asthma [43,44,45]. In addition, genetic risk factors can play an important role. Since 1989, more than 600 candidate genes have been described with respect to asthma [25].

Until today, there have not been many strategies for the prevention of asthma development. However, some targeted strategies are already being implemented. There are studies such as the MAKI study, in which healthy pre-term infants (32–35 weeks of gestation) who received Palivizumab for RSV immunoprophylaxis showed a reduced frequency of subsequent wheeze [46]. Another important point is early active tolerance induction, for which several studies showed that, e.g., desensitization against grass pollen led to a short-term reduction in asthma symptoms and decreased asthma medication. However, there were no effects on long-term asthma incidence [47]. Other preventive strategies are still in the experimental stage. Biological factors such as monoclonal antibodies targeting type 2 immunity show remarkable therapeutic effects and are widely used in addition to conventional therapies [48,49]. Today, clinical trials such as the Preventing Asthma in High-Risk Kids (PARK) study (NCT02570984) investigate the suitability of treatment with biological factors for prevention. Another potentially promising approach is microbial interventions. The ORak Bacterial Extract (ORBEX) trial (NCT02148796) studies whether oral bacterial lysates (OM-85) alter the outcome in high-risk children.

There are additional, untargeted strategies, which could support asthma prevention. One is a ban on smoking in public places, implemented in more and more countries. In a review by Frazer et al., data from 21 countries showed reduced mortality for smoking-related illnesses and an overall positive impact of national smoking bans on improving cardiovascular and respiratory health outcomes [50]. Additionally, in Denmark, it has been shown that vitamin D3 supplementation during pregnancy led to a reduced number of preschool wheezing episodes in children [42,51].

## 3. *Staphylococcus aureus (S. aureus)*

The first description of *S. aureus* is from 1880 by Alexander Ogston who was able to detect this pathogen in purulent abscesses. The bacterium owes its name to its characteristic cluster-like aggregation (*staphyle*, Greek for “grape”) and the golden yellow pigmentation of its colonies (*aurum*, Latin for “gold”) [52]. *S. aureus* is a Gram-positive facultative bacterial pathogen, which can be distinguished from other, less pathogenic *staphylococci* by its ability for β-hemolysis and positive mannitol-, fermentation-, and desoxyribonuclease-tests [53,54]. As a commensal, *S. aureus* constantly colonizes approximately 30% of the adult population [55,56,57]. Preferred sites of colonization are the skin and mucous membranes of the nasopharynx [55,58,59,60]. Newborns tend to show higher colonization rates of more than 70%. However, the colonization rate declines with increasing age so only 21% of six-month-old babies tested positive for *S. aureus* [55]. Some population groups tend to show more frequent colonization with *S. aureus* (up to 80%), e.g., healthcare workers, patients with type 1 diabetes, people using intravenous drugs, hemodialysis patients, surgical patients, and patients with acquired immune deficiencies and atopic diseases [52,54]. Colonization with *S. aureus* can be constant or intermittent, but not all studies on *S. aureus* colonization take this into account [61].

In addition to its role as a commensal, *S. aureus* can cause several diseases, which can range from deep skin infections to life-threatening conditions such as pneumonia or sepsis [55,62]. Antibiotic resistance complicates the treatment of *S. aureus* infections. Approximately 60% of all *S. aureus* isolates express a β-lactamase and are therefore resistant to β-lactam antibiotics, e.g., ampicillin. Currently, 20% of all hospital isolates and 0.3% of colonizing isolates already show resistance to β-lactam antibiotics of the second generation, e.g., methicillin [63,64]. One reason for the rapid development of resistance is the highly variable genome of *S. aureus*, which is approximately 2.8 Mb in size. The genome difference between the two strains can be up to 20% [65,66]. The enormous variability of the genomes between different *S. aureus* strains is based on a large number of mobile genetic elements (MGE), which are used for horizontal transfer between two strains. MGEs contain pathogenicity islands, plasmids, transposons, prophages, and chromosomal cassettes [67,68]. As a result, MGEs contribute significantly to the spread of antibiotic resistance and determine the virulence of an *S. aureus* strain [69]. The most important virulence factors include superantigens (Sags), pore-forming units, and exfoliative toxins. *S. aureus* SAgs include, for example, staphylococcal enterotoxins (SEs), enterotoxin-like toxins, and the toxic shock syndrome toxin 1 (TSST-1) [70].

Immunologically relevant host genes have been studied in *S. aureus* carriage and disease [71,72,73]. However, the identification of genetic risk (or protective) factors stands at its beginning, and it remains unclear whether such factors potentially even overlap with those associated with asthma or asthma severity. Further, despite intensive efforts in recent decades, attempts to develop an *S. aureus* vaccine for humans have failed as no protective immunity could be generated [74,75,76,77].

## 4. *S. aureus* and Atopy

As mentioned, patients with atopic diseases such as atopic dermatitis (AD), allergic rhinitis (AR), and allergic asthma, are more likely to be colonized with *S. aureus*. In addition, *S. aureus* is capable of escaping the immune system through the induction of type 2 inflammation [78,79].

AD is a chronic and relapsing inflammatory disorder of the skin affecting mainly infants and young children but also occurring during adulthood [80]. It is characterized by an impaired epidermal barrier function, dysbiosis of the cutaneous microbiota, and recurring infections, especially with *S. aureus* [81,82,83,84,85,86]. The skin of up to 100% of patients with AD is colonized with *S. aureus* from which up to 65% of isolates were found to be able to produce enterotoxins with superantigenic properties, e.g., staphylococcal enterotoxin B (SEB) [87,88].

AR is an inflammatory condition caused by an IgE-mediated type 1 hypersensitivity response to a variety of environmental allergens [89,90]. It is characterized by nasal congestion, anterior and posterior rhinorrhea, itching of the nose, and sneezing for more than one hour on two or more consecutive days [91]. As in patients with AD, *S. aureus* colonization rates in patients with AR are described to be significantly higher than in healthy individuals [92,93,94,95].

Allergic asthma is one of the common comorbidities associated with AR and it has been shown that 21–27% of patients suffering from allergic asthma in combination with AR were IgE-sensitized against staphylococcal toxins such as staphylococcal enterotoxin A (SEA), SEB, or TSST-1 [96]. For severe asthmatics, positive associations between SEB-IgE-sensitization and comorbidities (chronic rhinosinusitis (CRS) and CRS with nasal polyps (CRSwNP)) were recently described. In that study, disease onset was, on average, earlier in patients with SEB-IgE, while there was, however, no significant association with atopy [97]. Moreover, in severe asthma, a positive association between SEB-IgE and AD has been described [98]. For allergic asthma, significant relationships between *S. aureus* nasal carriage and the disease have been recognized, and allergic sensitization against staphylococcal enterotoxins, mainly SEB, is observed [34,37,99,100,101]. In addition to SEB, other *S. aureus*-derived proteins such as the serine protease-like protein D (SplD) have been shown to support Th2-biased immune responses after intratracheal (i.t.) exposure [102,103]. While nasal *S. aureus* colonization itself has a significant relationship with asthma prevalence [34], data with respect to correlations with asthma severity are clearer for sensitization against its toxins.

These findings indicate that *S. aureus*, in addition to its primary role as a colonizer and facultative pathogen, could also play an important role in allergic diseases. However, to date, it is not clear by which mechanisms *S. aureus* acts on atopy and how atopy in turn possibly supports colonization.

## 5. Pathophysiology of Allergic Asthma and How It Is Affected by *S. aureus*

Th1 cells play an important role in the protection against intracellular pathogens and are responsible for the induction of phagocytosis by, e.g., macrophages or dendritic cells (DCs). Additionally, due to their specific cytokine production, B cells are activated and produce complement-retaining and opsonizing antibodies. In contrast, Th2 cells ensure protection against helminths and promote acute and chronic inflammatory responses against allergens. The clearance of extracellular pathogens and fungi is the main role of Th17 cells [104]. During allergic responses, the balance between Th1, Th2, and Th17 responses is shifted towards a type 2 or Th2-dominated response. Due to the secretion of a large amount of type 2-specific cytokines, Th1 and Th17 cells are suppressed, resulting in a potentiating type 2 immune response. This in turn leads to allergic inflammation in which a large number of immunological processes take place at the same time [105]. In these, structural cells, cells of the innate and adaptive immune systems, and a variety of humoral factors are involved. In many points, however, it remains unclear which factors are responsible for the shift towards Th2-responses in atopic individuals.

### 5.1. The Airway Epithelium

According to the American Academy of Allergy, Asthma, and Immunology (AAAAI), “an allergy is a chronic condition involving an abnormal reaction to an ordinarily harmless substance called an allergen. Allergens can include aeroallergens such as dust mite, mold and tree weed and grass pollen, as well as food allergens such as milk, egg, soy, wheat, nut or fish proteins” [106].

In type 2 asthma, different soluble mediators and cell types are of crucial importance. Airway epithelial cells form the first barrier for inhaled allergens and pathogens, and it has been shown that allergens such as house dust mites (HDMs) or cockroach antigens can lead to a loss of epithelial cell–cell contacts due to their enzymatic activities [107]. Due to the fact that epithelial cells express a myriad of innate pattern-recognition receptors, such as Toll-like receptors (TLRs), nucleotide-binding oligomerization domain (NOD)-like receptors, retinoic acid-inducible gene-(RIG)-I-like receptors, C-type lectin receptors (CLRs), and others, they are able to respond to a variety of external triggers by producing cytokines and chemokines [17]. In mice, allergen exposure is able to trigger the production of epithelial-derived cytokines, such as IL-1α, transforming growth factor β (TGF-β), or granulocyte-macrophage colony-stimulating factor (GM-CFS) [108,109]. The most extensively studied cytokines in this context are the epithelial alarmins IL-33, thymic stromal lymphopoietin (TSLP), and IL-25, which contribute to type 2-high asthma [110,111].

Patients with allergic eosinophilic asthma show increased levels of serum IL-33 as compared to non-eosinophilic phenotypes [112], and higher levels of IL-25 were associated with greater airway hyperresponsiveness, increased airway and blood eosinophils, and higher levels of serum IgE [113]. Furthermore, levels of the epithelial-cell-derived cytokine TSLP correlate with disease severity, glucocorticoid resistance, and airway obstruction in asthmatic patients [114,115]. Furthermore, different groups showed that TSLP is able to mediate interactions between airway structural cells, e.g., bronchial smooth muscle cells, and immune cells, e.g., mast cells, [116] or promote immune cell differentiation under Th2-polarizing conditions [117]. Tezepelumab is a human monoclonal antibody that specifically binds TSLP and thereby blocks its interaction with its heterodimeric receptor [118]. Several phase-three clinical studies have shown that treatment with Tezepelumab significantly reduced asthma exacerbations and improved asthma control, lung function, and health-related quality of life [115,119]. Tezepelumab was authorized for the treatment of patients with severe, uncontrolled asthma in December 2021 in the US. In the EU, the authorization for treatment was confirmed in September 2022, underlining the key role of epithelial cytokines in asthma pathogenesis.

Several in vitro studies with human epithelial cell lines have shown that *S. aureus*, as well as some of its proteins, is able to induce inflammatory responses in airway epithelial cells. For example, increased IL-8 concentrations were detected in the supernatant of human airway epithelial cells treated with non-toxic concentrations of staphylococcal enterotoxins A and B (SEA, SEB) after 24 h [120]. As a chemotactic factor, IL-8 attracts neutrophils, basophils as well as T cells during inflammatory processes. Another study showed that in vitro S. aureus-stimulated airway epithelial cells produced significantly increased levels of RANTES, IP-10 and eotaxin [121]. Those chemokines altogether also attract immune cells to inflammatory sites and thereby drive inflammatory processes. In addition, S. aureus can induce the production of the typical epithelial cytokines TSLP and IL-33 in human mucosal tissue [122]. In summary, these studies indicate S. aureus to potentially induce or intensify inflammatory and allergic responses when colonizing the human respiratory mucosa and thereby contribute to the development of allergic asthma.

### 5.2. Effector Cells and Soluble Mediators in Allergic Asthma

Following respiratory epithelial cells as first contact, DCs are next in line in allergen-directed immune responses. During allergic sensitization in allergic asthma, inhaled allergens are taken up and processed by antigen-presenting cells (APCs), particularly DCs. These are essential for initiating the specific allergen-directed immune response, i.e., sensitization, and for the induction of allergic airway inflammation (AAI). They migrate to the draining lymph nodes, where the processed allergen is presented to antigen-specific B and T cells in the form of peptide antigens [123,124]. Most conventional DCs found in the lungs of asthma patients express the high-affinity IgE receptor FcεRI, which indicates a role of IgE and DCs in Th2 airway inflammation [125,126]. It has further been shown that DCs contribute to the allergic reaction in sensitized individuals by amplifying the Th2 cell response [127,128,129].

*S. aureus* is able to affect the physiological functions of DCs in various ways (reviewed in [130]). For example, the secretion of staphylococcal superantigen-like proteins (SSL) 3 and 4 diminishes its recognition by DCs via TLR2, as these proteins are able to interfere with lipopeptide binding and TLR2 dimerization [131]. To avoid phagocytosis, *S. aureus* is able to invade different cell types utilizing adhesins [132,133,134]. Furthermore, *S. aureus* is able to produce leukocidins, which can kill host immune cells, including DCs [135].

Following antigen presentation to T and B cells in the local draining lymph nodes, the resulting interaction between APC (i.e., DCs) and the lymphocyte induces specific responses. These depend on and are shaped by the cytokine milieu and the presence or absence of specific co-stimulatory molecules. Due to peptide presentation via major histocompatibility complex-II (MHCII) molecules on the surface of DCs and under the influence of epithelial-derived cytokines, allergen-specific Th2 cells are activated [136]. This activation leads to a massive clonal expansion of Th2 cells, which are crucial for the induction of asthma symptoms. In an ovalbumin (OVA)-induced mouse model for AAI, it has been shown that the depletion of CD4^+^ T helper cells prevented asthma development. At the same time, the adoptive transfer of in vitro-polarized Th2 cells from mice with transgenic expression of an OVA peptide-specific T cell receptor led to features of AAI including a strong production of specific type 2 cytokines such as IL-4, IL-5, and IL-13 [137].

Patients with acquired or inborn T cell deficiencies, such as HIV patients, are particularly affected by *S. aureus* colonization and infections [138]. In addition, patients with genetic defects in Th17 cell development are highly susceptible to *S. aureus* infections [139,140]. This indicates that T cells are strongly involved in protective immune responses to *S. aureus* infections and contribute to their clearance. As important as T cells are for fighting the infection, they may also contribute to noxious inflammatory reactions. *S. aureus* is able to produce superantigens. Already small concentrations are able to unspecifically activate a high number of T cells leading to massive cytokine production. Such a cytokine storm can result in toxic shock and organ damage [141,142].

Type 2 innate lymphoid cells (ILC2s) display an additional cell type of the innate immune system that can be responsible for increased levels of type 2 cytokines. While transcriptionally and functionally mirroring Th2 cells, they do not express rearranged antigen receptors. In the pathophysiology of allergic asthma, ILC2s respond to unspecific cell-derived factors such as IL-25, IL-33, and TSLP with the production of very large amounts of type 2 cytokines. This cytokine milieu might contribute to enhancing the adaptive type 2 immune response and the priming of Th2 cells [143,144]. In addition to their role in allergic airway inflammation, ILC2s seem to also contribute to other types of airway inflammation such as non-allergic eosinophilic airway inflammation. In patients with eosinophilic nasal polyps, significantly increased numbers of ILC2s were observed in the lung and peripheral blood [143]. In addition, murine studies of non-allergic eosinophilic asthma showed ILC2s are required to develop eosinophilia and airway hyperresponsiveness [145,146]. A very recent study using ILC2-deficient mice indeed demonstrated their exclusive role in eosinophil recruitment [147].

The Th2 cytokines IL-4 and IL-13 promote the class switch of activated B cells to IgE-producing plasma cells [15,148,149]. IgE antibodies circulate in the blood and eventually bind to the high-affinity IgE receptor FcεRI expressed on the surface of, amongst other cells, mast cells. These display another key effector cell type in the pathophysiology of allergic asthma [150]. Once antigen-specific IgE molecules are cross-linked on the surface of mast cells following allergen exposure, mast cells will be activated, degranulate, and release a variety of soluble mediators such as histamine, leukotrienes, proteases, and prostaglandins [150,151]. Increased concentrations of these mediators lead to vasodilation, the contraction of smooth muscle cells, and mucus secretion and thereby promote inflammation [152,153]. Omalizumab is a recombinant, humanized monoclonal antibody that selectively binds to the Cε3 domain of IgE antibodies and blocks their binding to effector cells. Thereby, IgE-mediated activation and the release of cellular mediators are prevented [154,155]. In the US, Omalizumab was approved for the treatment of moderate to severe allergic asthma in 2003, and in Germany, patients with moderate and severe allergic asthma have been treated since 2005. Omalizumab therapy is indicated if patients have several severe asthma attacks a year despite treatment with a long-acting β2-sympathomimetic combined with high-dose inhaled glucocorticoids [156]. Several clinical trials demonstrated the efficacy of Omalizumab treatment with a reduced frequency of exacerbations, a reduction in inhaled corticosteroids, and statistical improvements in the quality of life in children, adolescents, and adults (reviewed in [157,158]). To date, the mechanistic contribution of *S. aureus*-specific IgE to the pathophysiology of allergic asthma remains unclear in many points. In principle, however, IgE-targeting therapies would also target *S. aureus*-specific IgE, with potential implications not only for systemic but also for local IgE in the airways [159].

In addition to class switching in B cells, there are additional functions of IL-4, such as promoting Th2 cell polarization, mucus production, and the transmigration of eosinophils across the endothelium, as well as mediating the expression of vascular cell adhesion molecule-1 (VCAM-1) [160,161]. IL-13 also has a broad spectrum of functions in asthma. Amongst other effects, IL-13 promotes the migration of eosinophils into the lungs by increasing the synthesis of eotaxin, induces goblet cell hyperplasia and thereby increases mucus production, leads to the proliferation of smooth muscle cells, and stimulates airway hyperresponsiveness [162,163,164,165,166]. Dupilumab, a recombinant monoclonal antibody specific for the type-I (IL-4Rα/γC) and type-II receptor (IL-4Rα/IL-13Rα), was recently approved for the treatment of AD, nasal polyposis, and asthma. It prevents the interaction of IL-4 and IL-13 with their receptors and thereby inhibits IL-4/IL-13 signaling. This simultaneous targeting of two mediators of type 2 inflammation can significantly decrease asthma exacerbation rates and improve lung function and respiratory symptoms [167,168,169].

IL-5 supports the recruitment and survival of one of the most important key effector cell types in AAI, i.e., eosinophilic granulocytes or eosinophils [170]. Eosinophils develop in the bone marrow and then circulate in the bloodstream. Direct correlations between eosinophil counts in the respiratory tract and asthma exacerbations have been demonstrated [171,172]. In addition to IL-5, other cytokines affect eosinophils. IL-3 and GM-CSF activate and enhance eosinophil functions, such as cytotoxic killing, superoxide, and leukotriene production [170,173]. Eosinophils only release their granules with cytotoxic, immune-modulating, and remodeling-promoting properties when they reach a target organ, such as the lungs in bronchial asthma, where they contribute to inflammation [174]. The released leukotrienes are potent bronchoconstrictors [175]. Furthermore, eosinophils regulate the immune response through direct effects on T cell functions, e.g., they influence both Th1 and Th2 cytokine production [176,177,178]. In humans, it also has been confirmed that eosinophils are able to present antigens to T cells [179]. There are several monoclonal antibodies, which act on IL-5-dependent eosinophil recruitment and/or deplete eosinophils through antibody-dependent cell-mediated cytotoxicity, three of which are currently approved for therapy of allergic asthma. Mepolizumab binds specifically to soluble IL-5 and thereby inhibits its interaction with its eosinophil surface receptor. Its efficacy has already been investigated in several phase-three trials demonstrating significantly decreased exacerbation rates [180,181,182]. Reslizumab also binds IL-5, and treatment with this monoclonal antibody also resulted in lower exacerbation rates and lower blood eosinophil counts [183,184,185]. Similar results have been achieved by Benralizumab. In contrast to Mepolizumab and Reslizumab, Benralizumab binds the IL-5 receptor-α on eosinophils and leads to their depletion [186,187,188]. *S. aureus* is potentially able to influence allergic inflammation by affecting eosinophil functions in multiple ways. In human eosinophil cultures, it has been observed that *S. aureus* induces eosinophil cell death through α-hemolysin [189], which would supposedly be beneficial. In contrast, another study showed that allergic inflammation is enhanced by *S. aureus* colonization due to *S. aureus*-induced induction of extracellular eosinophilic traps, which was associated with increased IL-5 secretion [190].

**Figure 1:**
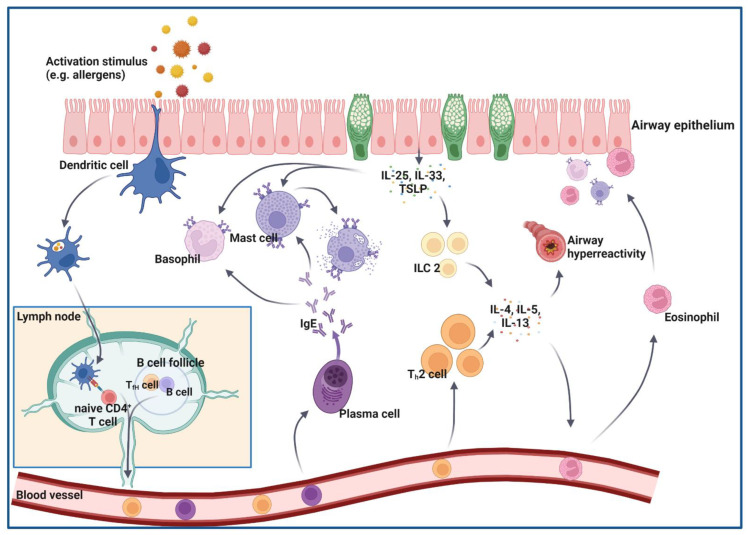
Key effector cells and soluble mediators in the pathogenesis of allergic asthma. After detection, internalization, and processing by dendritic cells, peptide fragments of the allergen are presented via MHII to naïve CD4^+^ T cells. These become activated, differentiate into Th2 cells, and produce huge amounts of typical type 2 cytokines, such as IL-4, IL-5, and IL-13. IL-4 and IL-13 promote class switching in B cells into IgE-producing plasma cells. Allergen-specific IgE antibodies bind to specific Fcε-receptors on the surfaces of basophils and mast cells. Following prior sensitization against a specific allergen, the respiratory tract is re-challenged with the allergen and allergen-specific Th2 cells are activated. Due to the cross-linking of membrane-bound, allergen-specific IgE antibodies by the allergen, the degranulation of basophils and mast cells is triggered, and mediators such as histamines or leukotrienes are released, further enhancing the allergic reaction. Blood eosinophils are recruited to the airway epithelium due to, amongst other chemoattractants, increased IL-5 concentrations. In addition to their barrier function, airway epithelial cells are able to produce a variety of cytokines, such as IL-25, IL-33 and thymic lymphopoeitin (TSLP), which further enhance the allergic reaction via the activation of basophils, mast cells, and innate lymphoid cells type 2 (ILC2). References are in the main text. Created with BioRender.com.

Figure 1 summarizes the key effector cells and soluble mediators in the pathogenesis of allergic asthma. Overall, the inflammatory pathways and mechanisms in patients with allergic asthma are very heterogeneous, resulting in varying symptoms and disease severity and a high demand for individual treatment regimens. The high heterogeneity also affects the efficacy of current asthma treatments. To ensure individual and fitting treatment, a central question in asthma research is the identification of asthma endotypes and specific drivers of inflammation.

### 5.3. Airway Hyperresponsiveness

Next to the cellular and humoral Th2 immune response in allergic asthma, the impairment of lung function parameters is a hallmark symptom and an important diagnostic parameter of this disease. Airway hyperresponsiveness (AHR) is a defining and consistent feature of bronchial asthma [191]. Over three decades ago, it was already shown that there are two semi-independent components to AHR:

(i) Persistent AHR is present in the majority of asthma patients. This component likely relates to physiological and structural changes in the airways, also known as airway remodeling, and reflects the chronicity of the disease.

(ii) Variable or episodic AHR is inducible by certain exposures or triggers and improved by others [192,193]. Different studies show that the variable component of AHR is associated with asthma activity and severity and at the same time reflects airway inflammation. To this day, however, the mechanisms underlying variable AHR remain largely elusive [191,194].

One of several parameters for the diagnosis of asthma is the measurement of AHR towards unspecific triggers with a methacholine provocation test. If it is known which specific allergen triggers the allergic asthma symptomatology, e.g., through a previous skin prick test or the analysis of specific serum IgE-levels, an allergen provocation test using this specific allergen can also be performed [195].

## 6. Staphylococcal Superantigens

### 6.1. Staphylococcal Enterotoxin B (SEB)

Staphylococcal enterotoxins are a family of 22 structurally related toxins discovered to date [196], and up to 80% of isolated *S. aureus* strains are capable of producing enterotoxins [101,197,198,199]. SEB is one of the best-characterized toxin in this family and is listed as a category B select agent by the U.S. Centers for Disease Control and Prevention [200]. It is resistant to heat, acids, and inactivation by gastrointestinal proteases such as papain, trypsin, or pepsin [198,201,202,203]. SEB belongs to the superantigen family of proteins produced by *S. aureus*, which are potent immune activators [204,205,206]. SAgs mediate a direct interaction between peptide-MHC class II and the CDR2 loop of the variable chain of the T cell receptor [207]. SAg-mediated T cell activation is therefore independent of antigen presentation on the MHCII molecule of APCs. At picomolar concentrations, SEB already leads to the activation of a very large fraction (5–30%) of the exposed T cell population [208,209,210]. Conventional antigens administered in a much higher concentration only activate approximately <0.01% of the exposed T cells [211]. Therefore, superantigens can lead to a cytokine storm, potentially resulting in toxic shock syndrome, multi-organ failure, and even death [212]. In addition to their function as superantigens, staphylococcal enterotoxins can also function as allergens. A recent study showed, via a basophil activation test, that almost 40% of patients suffering from severe asthma showed a positive basophil activation test for at least one enterotoxin. This finding further supports the important role of *S. aureus* and especially IgE responses toward its toxins in the pathogenesis of asthma [213].

In humans, the ingestion of less than 1 µg SEB can lead to food poisoning, which is characterized by symptoms such as nausea, vomiting, abdominal pain, cramps, and diarrhea [201,214]. In patients with AD, the severity of the disease correlates with the amount of colonizing *S. aureus* strains able to produce enterotoxins. Here, SEB induces the maturation of DC via TLR2, which then favors the polarization of naïve T cells to Th2 cells [215].

In mice, intraperitoneal (i.p.) administration of SEB leads to toxic shock, which manifests in increased levels of pro-inflammatory cytokines such as IL-1, IL-3, TNF-α, and IFN-γ [216,217]. When SEB is administered intranasally (i.n.) or via inhalation, it can trigger acute lung injury, characterized by excessive cytokine production, immune cell infiltration, necrosis in endothelial cells, and pulmonary edema [218,219]. Because of the lower toxin affinity to murine MHCII molecules as compared to human MHCII, it is necessary to use non-physiological doses of SEB or potentiating agents such as D-galactosamine or lipopolysaccharide (LPS), to investigate SEB-mediated effects in mice [211]. So far, few studies have experimentally addressed the short-term effects of *S. aureus* and SEB in the respiratory tract and on allergic asthma. In the serum of patients with severe asthma, SE-specific IgE antibodies were detected more often as compared to patients suffering from less severe asthma [220,221]. In a different study, Schreiber et al. showed that enterotoxin-specific IgE-sensitization in patients with severe asthma was mainly against SEB [40].

Apart from SEB-specific IgE-sensitization, SEB potentially also affects sensitization and allergic reactions to common allergens through its activities as a toxin and superantigen. In a mouse model, it has been shown that i.n. application of SEB alone leads to increased numbers of lymphocytes, neutrophils, and eosinophils in bronchoalveolar lavage (BAL) in a dose-dependent manner [222]. Combined with i.n. OVA-treatment, SEB facilitated OVA-specific allergic sensitization and inflammation and its activity was described as “adjuvant-like” [223]. Furthermore, repeated i.n. SEB-treatment immediately before the allergic challenge has been described to lead to enhanced AAI in previously sensitized mice, characterized by increased immigration of eosinophils to the respiratory tract, as well as increased mRNA-levels of IL-4, IL-5, and eotaxin-1 [224]. Huvenne et al. combined i.n. OVA-treatment with the additional treatment with different *S. aureus* toxins (SEA, SEB, and TSST-1) and LPS. Only the combination of OVA and SEB led to an increased OVA-specific IgE response, an increased influx of eosinophils and lymphocytes into the respiratory tract, and increased production of typical type 2 cytokines [225]. Others have shown that even SEB treatment of injured skin together with epicutaneous OVA-sensitization, led to an increase in airway inflammation upon i.n. OVA-challenge [215]. In HLA-DR3 transgenic mice, which express the human MHC molecule DR3 on all APCs, as well as CD4^+^ T cells, the i.n. application of SEB led to an increased accumulation of macrophages, eosinophils, and neutrophils in the BAL, as well as an increased airway resistance after methacholine challenge [226]. In 2020, we showed that i.n. administration of SEB has the potential to modulate AAI in multiple ways, with either ameliorating or aggravating effects. This modulation highly depended on the dose of SEB administered, as well as on the time-point of administration. While the administration of a relatively low SEB-dose (50 ng SEB) during the allergic challenge aggravated allergic inflammation, the administration of a high dose of SEB (500 ng SEB) led to a shift in the immune response from a type 2 towards a type 1 response. The higher dose of SEB further ameliorated inflammation, if administered before sensitization [227].

Taken together, clinical studies show significant correlations between the presence of *S. aureus* toxin-specific IgE and asthma severity, and experimental studies suggest a high immune-modulatory potential of SEB in AAI. Detailed knowledge of the underlying mechanisms will be essential for developing related diagnostic, prophylactic, and therapeutic approaches such as the elimination of *S. aureus* colonization or targeting of toxin-specific IgE.

### 6.2. Toxic Shock Syndrome Toxin 1 (TSST-1)

Another superantigen produced by *S. aureus* is the toxic shock syndrome toxin 1 (TSST-1). In addition to SEB-specific IgE-sensitization, 57% of patients with atopic dermatitis showed TSST-1-specific IgE antibodies in their serum, indicating a type 2 immune response against this toxin [228]. An in vitro study with isolated PBMCs from patients with AD showed that TSST-1 is able to enhance the IgE response against allergens [229]. A different study observed a significantly higher proportion of patients with AR alone and a combination of allergic asthma and rhinitis to show specific allergic sensitization against TSST-1 as compared to healthy controls. However, at the same time, it was shown that blood eosinophil counts did not correlate with serum IgE-levels specific for TSST-1 [96].

Furthermore, TSST-1 has a role in B cell hyperactivity, autoimmunity, and allergy. In isolated PBMCs, it was shown that while stimulation with a high dose of TSST-1 diminished the Ig production of B cells, it was significantly increased after stimulation with a low dose of TSST-1 [230]. Even though data on TSST-1 and atopy are scarce, these results indicate that, in addition to SEB, TSST-1 might also contribute to the development of inflammation in atopic diseases.

## 7. Serine Protease-Like Proteases (Spls)

Serine protease-like proteases (Spls) are a group of six proteases (SplA—F) secreted by *S. aureus*, the function of which has been unknown until today [231]. It has been shown that nasal polyp tissue of patients colonized with *S. aureus* contains IgG specific for SplA, SplB, and SplD/F. Additionally, asthma patients showed significantly higher serum titers of IgE antibodies specific for SplA, SplB, SplD, and SplE, suggesting a role of Spls in the pathogenesis of asthma [96,232]. Furthermore, it has been shown that the human T cell response to Spls differed from the IFN-γ/IL-17-dominated response elicited by other *S. aureus* antigens. Human T cells respond to Spls with an IL-4/IL-5/IL-10-response, which underlines their possible role in atopy [232,233]. SplD especially shows the potential to be involved in the pathogenesis of atopic diseases, as in the airways of patients with CRSwNP, significant amounts of SplD were detectable [234]. An experimental study showed the atopic potential of SplD in mice. When repeatedly administered i.t., SplD led to a significant production of IL-33 in the lungs. Furthermore, eotaxin production and eosinophil and ILC2 infiltration, goblet cell hyperplasia, and bronchial hyperreactivity increased, all of which are hallmark features of allergic asthma. It has been shown that SplD mediates an IL-33-dependent sensitization to OVA and that neutralizing IL-33 by soluble ST2 (IL-33 receptor) efficiently blocks SplD-induced allergic airway inflammation [103].

## 8. *Staphylococcus aureus* Protein A (SpA)

SpA is a 45kDa, multi-domain protein produced by almost all *S. aureus* strains, with a multitude of functions in the interaction with the host. It is anchored in the bacterial cell wall and released by proteolytic cleavage [235]. In contrast to the “conventional” superantigens produced by *S. aureus*, which mediate the direct interaction between MHCII and the T cell receptor, SpA is known for its ability to directly bind to the Fc-domain of human IgG, thereby interfering with the B cell receptor. SpA is referred to as a B cell superantigen because it can mediate a crosslinking between B cell receptors with V_H_3 elements [236]. In patients with CRwNP it has been shown that SpA can induce strong local IgE production in V_H_3-positive B cells [237,238,239]. In addition to the potential role in atopy, in mice, active vaccination studies with SpA or passively transferred SpA-neutralizing antibodies showed promising results. The antibody response to a broad spectrum of *S. aureus* virulence factors increased, and the clearance of colonization and protection against infection improved [240,241].

## 9. Methods

To identify relevant published studies, we searched the PubMed and Google Scholar databases. The keywords used were, amongst others, allergic asthma, allergic asthma pathophysiology, allergic asthma risk factors and prevention, allergic airway inflammation, *Staphylococcus aureus*, *Staphylococcus aureus* allergens, *Staphylococcus aureus* atopy, staphylococcal enterotoxins, *Staphylococcus aureus* enterotoxin B, Toxic-shock-syndrome toxin 1, serine-protease like proteins, and *Staphylococcus aureus* Protein A. The search for different keywords on PubMed returned between 70 and 43,000 results. The search on Google Scholar resulted in far more results (depending on the keyword, up to 2 million results). The retrieved publications were thoroughly and critically screened considering the publication date, relevance/focus, and the number of citations. The major results and findings of all 242 published articles cited, containing 109 original studies, 107 review articles, 18 reports of clinical studies, 4 books, and 4 guidelines, were summarized and discussed in this review.

## 10. Conclusions and Future Directions

**Figure 2:**
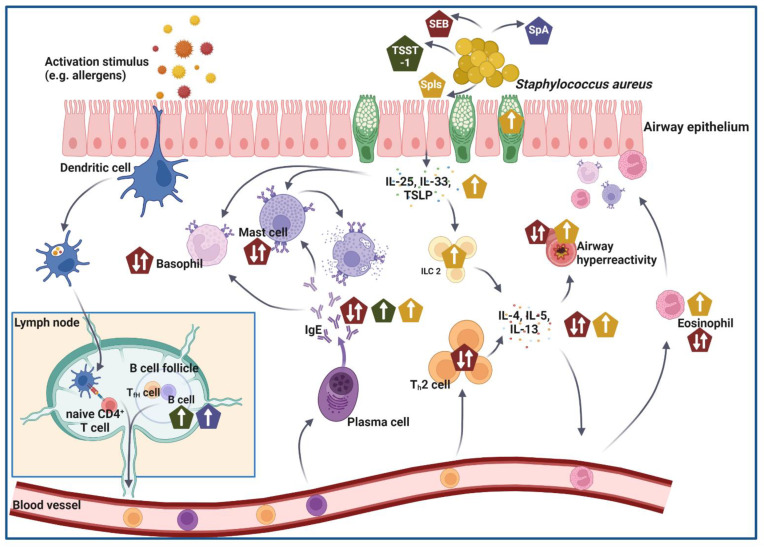
Processes in the pathogenesis of allergic asthma affected by different toxins produced by *S. aureus*. *S. aureus* is known for its ability to produce a variety of proteins, including several toxins. Belonging to the family of superantigens produced by *S. aureus*, the toxic shock syndrome toxin-1 (TSST-1; green pentagon) mainly affects B cell immunity by enhancing the number of B cells and thereby the amount of produced IgE antibodies (indicated by white arrows in green pentagons). Another member of the superantigen family, the *S. aureus* enterotoxin B (SEB; red pentagon) is presumably able to affect pathogenesis in multiple ways. In human studies, asthmatic individuals showed increased SEB-specific IgE-levels, as well as stronger asthmatic symptoms. In murine studies, it has been shown that intranasal SEB-administration can have either ameliorating or aggravating effects on allergic airway inflammation and affects various key effector cells and mediators involved in pathogenesis. White arrows in red pentagons indicate SEB-mediated effects. Serine-protease-like proteases (Spls; yellow pentagon) are a family of six proteases produced by *S. aureus*, which could also affect the pathogenesis of allergic asthma. In asthmatic patients, significantly higher Spl-specific IgE-levels have been observed, and in a murine study, it has been shown that SplD is especially able to induce an asthma-like phenotype after intratracheal administration (indicated by white arrows in yellow pentagons). The *S. aureus* protein A (SpA; blue pentagons) is referred to as a B cell superantigen, and in atopic patients, it triggers strong B cell proliferation and increased production of IgE antibodies. Those effects mediated by SpA are indicated through white arrows in blue pentagons. References in the main text. Created with BioRender.com.

In summary, research over the last decade has shown that *S. aureus* colonization and toxins produced by *S. aureus* play a major role in atopic diseases, especially allergic asthma and its pathophysiology. Figure 2 summarizes the processes in the pathogenesis of allergic asthma affected by different toxins produced by *S. aureus* and discussed in this review. Several key effectors in the pathogenesis of allergic asthma can be directly affected by *S. aureus*. Starting from inducing inflammatory processes in airway epithelial cells that potentially enhance the allergic reaction, *S. aureus* is further capable of affecting DC and eosinophil functions in multiple ways. In addition, with its ability to produce superantigens, *S. aureus* also potentially targets T cells in allergic asthma. Thereby, *S. aureus* can affect both the onset as well as the progression of the disease. Significant correlations between *S. aureus*-specific IgE-sensitization and asthma severity exist. However, the significance and the details of the underlying mechanisms remain largely elusive and need to be investigated further. On the one hand, it remains unclear when sensitization to *S. aureus* toxins occurs in one’s lifetime and whether the respiratory tract, skin, or alternative sites are decisive. Equally important, future research will have to define the functional role of such sensitization in atopy, i.e., whether and how *S. aureus* acts as a relevant allergen in IgE-sensitized patients. Further, detailed knowledge of the contribution of *S. aureus* toxins to the inflammation, in addition to IgE-sensitization as well as the immunological role of *S. aureus* colonization itself, will increase our mechanistic understanding of its interplay with allergic airway inflammation.
